# Grammars Across Time Analyzed (GATA): a dataset of 52 languages

**DOI:** 10.1038/s41597-023-02659-1

**Published:** 2023-11-28

**Authors:** Frederic Blum, Carlos Barrientos, Adriano Ingunza, Damián E. Blasi, Roberto Zariquiey

**Affiliations:** 1 Department for Linguistic and Cultural Evolution, Max-Planck Institute for Evolutionary Anthropology, Leipzig, Germany; 2 Institut für Linguistik, Universität Leipzig, Leipzig, Germany; 3 Pontificia Universidad Católica del Perú, Lima, Peru; 4 Department of Human Evolutionary Biology, Harvard University, Cambridge, USA; 5 Harvard Data Science Initiative, Harvard University, Cambridge, USA; 6 Center for Brain and Cognition, Pompeu Fabra University, Barcelona, Spain; 7 Catalan Institute for Research and Advanced Studies (ICREA), Barcelona, Spain

**Keywords:** Interdisciplinary studies, History

## Abstract

Grammars Across Time Analyzed (GATA) is a resource capturing two snapshots of the grammatical structure of a diverse range of languages separated in time, aimed at furthering research on historical linguistics, language evolution, and cultural change. GATA comprises grammatical information on 52 diverse languages across all continents, featuring morphological, syntactic, and phonological information based on published grammars of the same language at two different time points. Here we introduce the coding scheme and design features of GATA, and we describe some salient patterns related to language change and the coverage of grammatical descriptions over time.

## Background & Summary

There are approximately 6500 mutually unintelligible languages in the world^1^. Their varied social, ecological, and cultural setups have allowed us to explore fundamental questions about language and its relation to other domains of the study of humans. The world’s linguistic diversity constitutes a unique resource for understanding the cognitive basis of the human capacity to learn and use languages (e.g.^2–4^), for untangling human history at a global and regional scale (e.g.^5,6^), and for making inferences about language diversification and change (e.g.^7^). Contemporary approaches to the study of linguistic diversity rely extensively on databases with information about hundreds and even thousands of languages^7–10^. However, most of these databases display information about individual languages either at specific points in their history, or -more problematically- by combining reference sources from different points in time. This limits the study of dynamic processes of language change, as indirect inferences about the past history of languages need to be supplemented (for instance through phylogenetic histories^11^). Grammars Across Time Analyzed (GATA) is a novel resource that aims at full-filling the need for diachronic information about languages based on published descriptions of the world’s languages. GATA includes information for 52 diverse languages through the independent coding of two (or more) grammatical descriptions of the same language in different points of their histories.

The study and research on language change is of foremost importance across human sciences. Naturally, language change is the main source of information in historical linguistics, as it informs us about the biases, tempo, and dynamics of the linguistic system. GATA allows the exploration of fundamental questions in the field, including e.g. the speed of grammatical change^12^ and the presumed co-evolutionary processes that dominate language change^9^. Yet, more broadly, language change can be put in relation to non-linguistic questions about the human mind, culture, and history. For instance, languages are transmitted along traditions, social structures, and genes, so tracing changes in one domain can inform about processes that have taken place elsewhere^6^. At the same time, language structures and their associated patterns of change might reflect specific societal and cultural pressures directly. For instance, languages that are adopted by a large and diverse community of speakers with different linguistic backgrounds have been claimed to change in the direction of simplified morphology^13^ (c.f.^14^). In a similar line, languages that are not transmitted to the newer generations have been claimed to undergo intense language change, typically resulting in a significant simplification and reduction of their grammatical inventories^15–18^. GATA offers a unique resource for testing these hypotheses and other related claims with relevant and adequately coded information on language change.

## Methods

### Sample creation

The main design principle of GATA is providing a diverse set of language histories based on published scholarship. For this purpose, we tap on a thorough collection of digitized literature covering over 37,000 digitized books and articles on descriptive linguistics^19^. The collection comprises: (1) out-of-copyright texts digitized by libraries, scientific societies, and Google books; (2) texts posted online with a license that allows them to be used for research; and (3) texts under publisher copyright where quotations of short extracts are legal. A listing of the collection can be accessed via the open-access bibliography Glottolog^20^. All the documents in this collection have been digitized into machine-readable text through ABBYY Finereader 14, an optical character recognition (OCR) software, using the metalanguage as the recognition language. This collection comprises some 12,000 grammatical descriptions^21^. Based on this collection, we assembled a sample of grammars which were selected following these criteria:

1. There are two accessible grammars of the same language (at least) 25 years apart from each other. This guarantees that there is minimally a generation between the two snapshots of the same language.
2. The languages were chosen evenly with respect to geographic and genealogical distribution. This is for the purpose of providing a balanced perspective of language dynamics across the widely varying circumstances of different regions of the world and their language families.

**Fig. 1:**
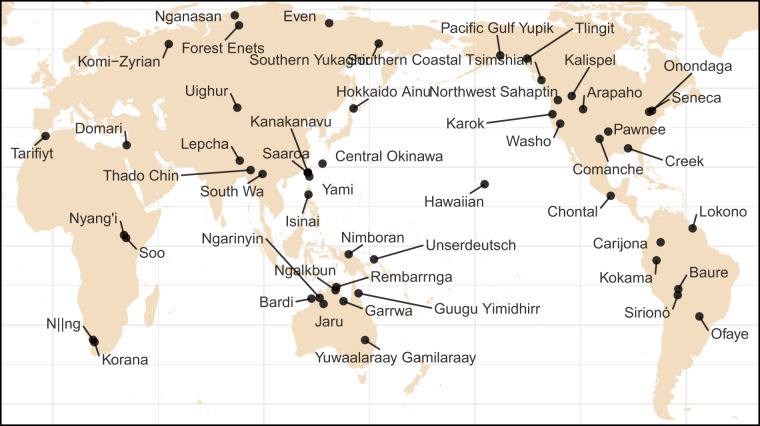
Approximate location of the languages included in the first release of GATA, based on Glottolog^1^.

Following these guidelines, GATA includes 52 languages coded for two reference times. Their geographic distribution can be observed in Fig. 1, and the time interval between the two grammars coded for each language is presented in Fig. 3.

### Features

We selected 31 grammatical features divided into six typological categories: grammatical relations, nominal categories, phonology, pronominal systems, verbal categories, and word order. Features are classified into three types: binary (b), numeric (n) and multi-state (m). They cover various grammatical domains ranging from phonology (e.g., number of vowels, consonants and tones) to morphology (e.g., number of cases, alignment types,tense-aspect-mood markers) and syntax (e.g., word order, interrogatives constructions, alignment types). More specifically, GATA includes 4 phonological features (n = 4), 18 morphological features (n = 7 and b = 11), and 9 syntactic features (m = 3 and b = 6). Table 1 lists all the grammatical features included in GATA.

**Table 1:** List of GATA features organized by domain.

Domain	Feature	Stability	Shortname	Type
Pronominal system	1 P pronoun distinctions	Relatively stable^33^	Pron1P	n
Pronominal system	2 P pronoun distinctions	Relatively stable^33^	Pron2P	n
Pronominal system	3 P pronoun distinctions	Relatively stable^33^	Pron3P	n
Nominal categories	Number of cases	Relatively stable^34^	Cases	n
Nominal categories	Spatial demonstratives	NA	Dem	n
Nominal categories	Numeral classifiers	Relatively unstable^34^	ClassNum	b
Nominal categories	Genitive classifiers	Relatively unstable^35^	ClassGen	b
Nominal categories	Grammatical gender	Relatively stable^33^	ClassNoun	b
Nominal categories	Instrumental vs comitative	Relatively stable^34^	InstrCom	b
Nominal categories	Alienable and inalienable possession	NA	Poss	b
Nominal categories	Locative vs directional	Possibly stable^34^	LocDir	b
Nominal categories	Temporal locative vs spatial locative	Possibly stable^34^	TempLoc	b
Nominal categories	Ditransitive argument marking	Possibly stable^34^	DitransMarking	b
Nominal categories	Agreement between noun and adjectives	NA	NP_Agr	b
Grammatical relations	Core arguments via case	Possibly unstable^34^	CA_case	b
Grammatical relations	Core arguments via head marking	Possibly unstable^34^	CA_head	b
Grammatical relations	Core arguments via word order	Possibly unstable^34^	CA_wordorder	b
Grammatical relations	Pronominal alignment	Possibly unstable^34^	PronAlign	b
Grammatical relations	Nominal alignment	Possibly unstable^34^	NomAlign	m
Verbal categories	Tense and aspect markers	NA	TA_marks	n
Verbal categories	Evidential markers	NA	Evid	n
Verbal categories	Interrogatives	NA	Questions	m
Verbal categories	Causative	Relatively unstable^33^	MorphCaus	b
Verbal categories	Agreement between verb and argument	Possibly unstable^33^	VP_Agr	b
Phonology	Number of consonants	NA	Consonants	n
Phonology	Number of tones	Stable^33,34^	Tones	n
Phonology	Number of oral vowels	NA	Vowels	n
Phonology	Number of nasal vowels	Stable^35,36^	NasVowels	n
Word order	Adj-N order	Relatively unstable^37^	AdjN	m
Word order	Gen-N order	Relatively stable^37^	GenN	m
Word order	Basic word order	Possibly stable (some)^33^	BasicWO	m

Shortnames and Type and references to illustrative claims on relative stability are also included.

The criteria for selecting GATA’s 31 grammatical features are twofold. Firstly, we included salient grammatical features whose presence/absence would be easy to determine from the description and/or the examples in each state of language (particularly in the older one, which may be associated with a relatively old grammatical description, which has not benefited from contemporary advances in descriptive linguistics). Thus, we avoid grammatical categories that have not been typically discussed until recently (such as applicative, mirative, frustrative, and engagement). Other categories, associated, for instance, with person, case, tense, vowels and consonants would be expected to show up even in more traditional grammars.

A second criterion for feature selection relates to the stability of the features^9^. We selected features that have been singled out, as being particularly labile for change (e.g., various types of classifier sets and the relative position of the adjective in relation to the noun), as well as others that have been claimed to exhibit extraordinary stability through time (e.g., gender markers, distinctions in first person pronouns, and case systems).

### Coding

The coding was carried on by a careful evaluation of the grammatical descriptions selected for each language, based on the collection described in the section on Sample creation. Each grammatical feature was coded for each of the two reference grammars resulting from our search, which we refer to as ‘states’. For each feature and each state, we included the following domains:

- Value. Introduces the data point for each feature, based on the typology presented in the Features subsection: binary (b), numeric (n) and multi-state (m).
- Reliability. Provides an assessment of the reliability of the original source in relation to the feature. Only for those cases in which the evidence was conclusive, (2) was coded, while non-conclusive evidence was coded with (1). (1) was mostly used for cases that presented a lack of coverage in the grammatical description, or for instances of explicit uncertainty expressed by the original author.
- Reference. The course of each data point is coded following the format author (year: page). A complete list of references is provided in the first release of GATA.
- Comments. Open-ended entry dedicated to relevant information not captured by the other fields.

Each language description underwent a detailed scrutiny. Three independent coders were assigned each other the grammars, and two senior researchers revised and curated their initial coding. The process involved a number of decisions in relation to the quality of grammatical descriptions as well as assumptions about unspoken conventions on grammar-making. We illustrate the nature of this process by highlight a handful of cases.

#### Illustrative cases

**Lokono** is an Arawakan language which received extensive documentation in the 19th century as well as a more recent description^22,23^. The author of the grammar corresponding to ‘state 1’ did not describe the Tense-aspect-mood (TAM) markers systematically or exhaustively, although evidence for TAM markers is present in glossed examples included in the grammar. Given this, we conclude that the author might have in fact missed altogether some TAM markers which are described in the ‘state 2’ grammar. This led us to code (1) ‘non-conclusive’ in the certainty column for ‘state 1’ in relation to TAM markers. The same author presents the notion of ‘letters’ instead of phonemes, a recurring issue in colonial documents, so no reliable inference regarding the phonological inventory of the language can be drawn either, which resulted in a further ‘non-conclusive’ judgment in relation to phonology.

**Central Okinawan** is a Japonic language with two grammatical descriptions^24,25^. The author of the earlier publication provides a list of personal pronouns, in which two sets based on politeness are proposed for the second and the third person. The author extensively discusses the pragmatic differences between various of these pronominal forms in a footnote. The total set of personal pronouns in Central Okinawan according to the author was 18 (including up to 12 third person pronouns with different honorific meanings). A value of (2) was assigned in the certainty column as the evidence seemed conclusive. In the more recent grammatical description of Central Okinawa, however, the author only documents two pronominal forms for the third person. The set of honorific pronouns in the third person paradigm seems to have disappeared in between both documents. For the two other person paradigms, in turn, the pronominal sets did not change significantly. The number of first person pronouns increased by one, while the number for second person pronouns remained the same. Despite the differences attested between the two grammatical descriptions, the latter one offers a detailed discussion of the pronominal set listing and illustrating all the attested free forms. We then coded a value of (2) in the certainty column for’state 2’ too.

**Kukama-Kukamiria** is a Tupi-Guaraní language spoken in Peru. For this language, there are two grammatical descriptions available: a textbook with abundant grammatical information^26^ and a contemporary reference grammar^27^. The first source does not incorporate any discussion on evidentiality, perhaps because this term was not widespread enough by the publication time of the source, and the markers that the more recent source describes as evidentials appear very superficially analyzed as modal markers. This led to a coding of evidentiality that assigns (1) ‘non-conclusive’ in the certainty column for the first source, and (2) ‘conclusive’ for the second one. A very similar situation is found regarding TAM markers. The older source describes only four tense suffixes, while the newer source lists eight clitics encoding both tense and aspect. It turned out that Kukama-Kukamiria aspect markers were also listed in the first source, but as independent words and not as bound morphemes. This may relate to the sometimes elusive morphosyntactic nature of clitics, which may manifest as dependent markers that exhibit phonological and prosodic properties of independent words. Clitics are somewhere in-between more clear-cut morphological categories like affixes and words. Thus, both sources have the same number of TAM markers, and the differences between the two states may be the result of a grammaticalization process, according to which independent words became grammatical clitics. Note that these difference may also be linked just to two distinct analysis on the morphological nature of the elements under discussion. It must be taken into consideration that the first source is one of the first studies of the language and was not oriented towards a linguistic audience, but rather to Spanish speakers who would like to learn the language. In turn, the second source is a contemporary functional oriented referential grammar. In the second source, one would expect a more detailed discussion of morphological elements in Kukama-Kukamiria. In any case, the data discussed here do not reveal a process of morphological reduction in association with the second stage, and both stages reveal basically the same number of forms.

The cases listed in this section show the importance of a careful qualitative analysis of the data, particularly in those instances where discrepancies between the two states of a language are identified.

## Data Records

The dataset is stored on Zenodo (10.5281/zenodo.8250217)^28^ and curated on Github (https://github.com/cldf-datasets/gata). The current release of the repository is Version 1.0.0 and was peer-reviewed in 2023. All data is available under a CC-BY 4.0 license. In total, the dataset contains 3224 observations across 52 languages. We present the two sources per language in Table 2. In order to make GATA maximally compatible with other cross-linguistic databases, we adopt the Cross-Linguistic Data Formats (CLDF)^29,30^. This framework supports sharing, re-use and comparison of data in a cross-linguistic framework. One of the central underlying principles of CLDF is the use of csvw-files. Instead of storing all the data in a single file, it is stored in separate but linked tables. For example, GATA is directly linked to Glottolog^20^, so that all languages are uniquely identified. This allows us to align our code and data with the FAIR^31^ principles: Findable, Accesible, Interoperable, and Reusable.

**Table 2:** List of the grammars sample per language and the time apart from each other.

Language	Grammar 1	Grammar 2	Time lapse
Arapaho	Sallzmann 1963^38^	Cowell and Moss 2008^39^	45
Bardi	Metcalfe 1972^40^	Bowern 2012^41^	40
Chontal	Waterhouse 1967^42^	O’Connor 2004^43^	37
Kukama-Kukamiria	Faust 1972^26^	Vallejos 2016^27^	44
Kalispel	Vogt 1940^44^	Speck 1977^45^	37
Karok	Bright 1957^46^	Halpern 1997^47^	40
Lepcha	Mainwaring 1876^48^	Plaisier 2003^49^	127
Ngarinyin	Coate and Oates 1970^50^	Spronck 2015^51^	45
Ofaye	Gudschinsky 1971^52^	De Oliveira 2006^53^	35
Onondaga	Zeisberger 1888^54^	Chafe 1970^55^	88
Seneca	Holmer 1954^56^	Chafe 2015^57^	51
Siriono	Schermair 1949^58^	Dahl 2014^59^	55
Uighur	Nadzhip 1971^60^	Yakup 2005^61^	34
Washo	Kroeber 1907^62^	Jacobsen 1964^63^	57
Yami	Asai 1936^64^	Rau and Dong 2006^65^	70
Baure	Adam and Leclerc 1880^66^	Danielsen 2007^67^	127
Central Okinawan	Loveless1963^24^	Miyara 2015^25^	52
Comanche	Pimentel 1865^68^	Charney 1993^69^	128
Creek	Nathan 1977^70^	Innes, Alezander and Tilkens 2004^71^	27
Garrrwa	Furby and Furby 1977^72^	Mushin 2012^73^	35
Guugu	Roth 1901^74^	Haviland 1979^75^	78
Hidatsa	Mathews 1965^76^	Park 2012^77^	47
Jaru	Tsunoda 1981^78^	Senge 2015^79^	34
Kanakanavu	Tsuchida 1975^80^	Wild 2018^81^	43
Nimboran	Anceaux 1965^82^	May 1997^83^	32
Sahaptin	Jacobs 1931^84^	Jansen 2010^85^	79
Pawnee	Parks 1976^86^	Cruickshanks 2011^87^	35
Rembarrnga	McKay 1975^88^	McKay 2000^89^	25
Saaroa	Tsuchida 1975^80^	Pan 2012^90^	37
Wa	Drage 1907^91^	Seng 2012^92^	105
Tarifiyt	Sarrionandia 1905^93^	McClelland 1996^94^	91
Thado	Krishian 1980^95^	Haokip 2014^96^	34
Yuwaalaraay	Williams 1980^97^	Giacon 2014^98^	34
Carijona	Koch-Grünberg 1908^99^	Moreno 2000^100^	92
Domari	Macalister 1914^101^	Matras 2012^102^	98
Even	Benzing 1955^103^	Kim 2011^104^	56
Forestenets	Castrén 1854^105^	Siegl 2013^106^	159
Hawaiian	Andrews 1854^107^	Elbert and Pukui 1979^108^	125
Hokkaido	Refsing 1986^109^	Bugaeva 2012^110^	26
Isinai	Scheerer 1918^111^	Perlawn 2015^112^	97
Komi	Von der Gabelentz 1841^113^	Hausenberg 1998^114^	157
Korana	Meinhof 1930^115^	Maingard 1962^116^	32
Lokono	Crevaux, Sagot and Adam 1882^22^	Pet 2011^64^	129
Ngalkbun	Capell 1962^117^	Cutfield 2011^118^	49
Nganasan	Castren 1966^105^	Wagner-Nagy 2019^119^	165
Niing	Maingard 1937^116^	Collins and Namaseb 2011^120^	74
Nyangi	Heine 1975^121^	Beer, McKinney and Kosma^122^	34
Southern Coastal Tsimishian	Boas 1911^123^	Dunn 1979^124^	68
Yukaghir	Jochelson 1905^125^	Maslova 1999^126^	94
Tiingit	Boas 1917^127^	Naish 1979^128^	62
Unserdeutsch	Volker 1983^129^	Leindensfelser and Maitz 2017^130^	34

The main folder of the dataset that is intended for re-use is ‘cldf/’, which consists of several linked csvw-files. As required by the CLDF model, our dataset has four central entities, each in its own file: Languages (‘languages.csv’), Parameters (‘parameters.csv’), Values (‘values.csv’), and Sources (‘sources.bib’). A descriptive JSON file (‘StructureDataset-metadata.json’) links the tables together while defining the relation between them. The ‘requirements.txt’ file indicates the necessary Python packages for reproducing the conversion into CLDF.

The ‘languages.csv’ file contains the columns ‘ID’, ‘Name’, ‘Macroarea’, and coordinates (‘Latitude’, ‘Longitude’) of each language. It also includes the ‘Glottocode’ as well as information on the endangerment status of the language (‘AES’). The ‘parameters.csv’ file stores the information about the 31 parameters of GATA. Each parameter is given an ‘ID’ and ‘Name’, as well as a ‘Shortname’. The ‘Description’ of the parameter is given in English and Spanish (‘Description_esp’). The 31 different parameters are sorted into six different linguistic categories (‘Category’, ‘Category_esp’). The columns ‘Shortname’ and ‘Variable_type’ contain information on how the parameter is coded in the data table. The ‘Comments’-column includes the instructions that were given to the coders for filling out the questionnaires

The final component of the data is stored in ‘cldf/values.csv’ and contains one observation per row. Each entry has its own ‘ID’. The columns ‘Language_ID’ and ‘Parameter_ID’ link the observation to the respective language and parameter. The value of the observation is given in the column ‘Value’. Further, all observations include the bibtex-key of the ‘Source’ (linked to ‘sources.bib’), the ‘Certainty’ of the observation, specific page-references (‘Reference’) for examples, and the ‘Year’ of the publication of the data. In some cases, the coders have added a ‘Comment’ to the observation, which provides further information on the judgments and assumptions that were made during the analysis.

We rigorously followed the workflow and examples provided by the CLDF documentation. This included the usage of the CLDFBench package^32^ to customize and create the dataset in CLDF automatically (see the Code availability section). The individual files in the repository are part of the CLDF workflow and describe the different contents, such as the cldfbench-script (‘cldfbench_gata.py’), the contributors table (‘Contributors.md’), the license, or the metadata. Dataset-specific files that were used in order to convert the data into CLDF are stored in the folders ‘etc/‘ and ‘raw/‘. Those two folders are only used for creating the CLDF dataset and should not be used as data source. The ‘raw/‘-directory contains the combined raw data (‘gata_raw.csv’), a bib-file with all sources, the original questionnaires, and all scripts that have been used during pre-processing. The ‘etc/‘-directory stores metadata that has been used during the conversion to CLDF. This includes a list of all parameters (‘parameters.csv’) as well as the list of languages (‘languages.csv’), incorporating also information on their vitality status. In addition to the standard CLDF folders, we also created a folder ‘plots/‘ which contains descriptive plots of the data and the code to create them.

## Technical Validation

We assess GATA in the light of general desiderata that apply to all cross-linguistic and cross-cultural databases: balanced sampling and feature coverage. In addition, we discuss the temporal distribution of the time gaps between grammars.

The **balanced sampling** design principle entails that, to the extent possible, the resource should provide an accurate perspective of the diversity and the variation present across the world’s languages. The current version of GATA^28^ comprises information on 52 languages, representing in a balanced manner, by design, all major linguistic regions in the world, as shown by the map presented in Fig. 1.

**Fig. 2:**
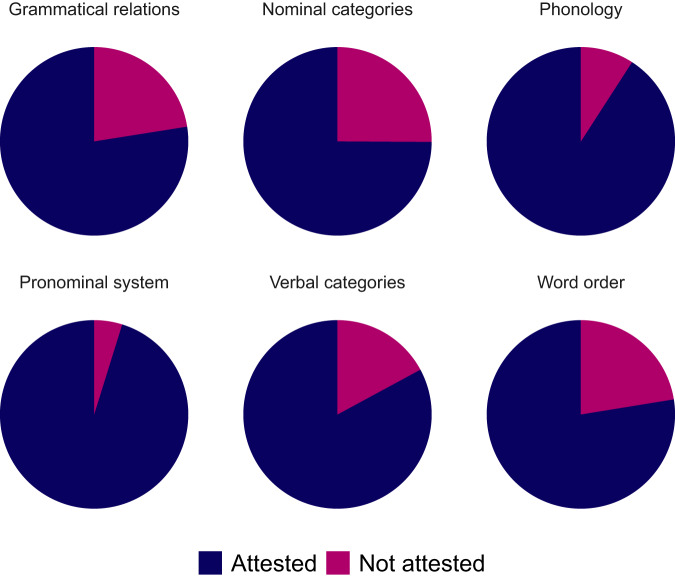
Feature coverage for each typological domain in GATA.

Reference grammars might not specify the features coded in GATA for a number of reasons, which would lead to uneven **feature coverage**. A visualization of the feature coverage for each typological domain is featured in Fig. 2, where we can see that no typological domain in GATA has a feature coverage under 75% of the total number of languages, being phonology and pronominal systems the better covered features with a coverage of over 90%.

**Fig. 3:**
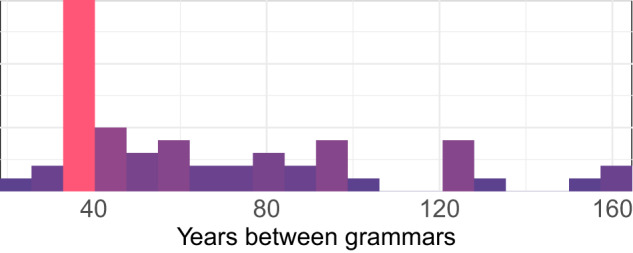
Time intervals between states (published grammars) in the languages coded in GATA.

The **temporal distribution** between the two states of a language coded in GATA varies significantly across languages, as observed in Fig. 3. One language in the sample, Rembarrnga, includes two states separated by 25 years, while there are three languages, Komi-Zyrian, Forest Enets, and Nganasan, for which the time interval between states is approximately 160 years (see Fig. 3).

**Fig. 4:**
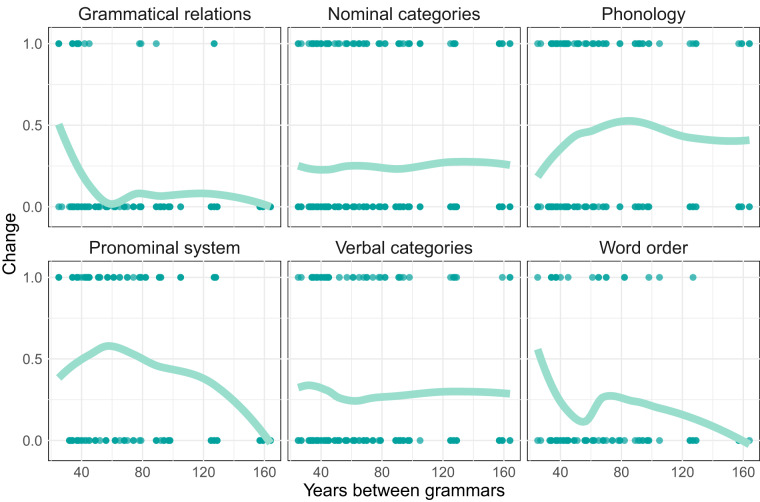
An example analysis of change across the different domains. The amount of change within each domain is plotted against the difference in years between both states of documentation.

Finally, we can assess the aggregated amount of language change emerging from each of these domains across time. Figure 4 showcases, within each grammatical domain, the fraction of all features who have changed over a given period of time. There is substantial variation in the total amount of change witnessed across domains, partially due to the differing temporal stability of linguistic features^9^. It should also be noted that, larger time intervals are not necessarily associated with larger amounts of language change - within the relatively narrow time span between the grammars analyzed in this paper. This can be understood as (partially) reflecting how language change is widely modulated by varying social and cultural factors, but it could point as well to a more subtle effect associated with the perceived utility of grammars. In a nutshell: given that a grammar of a language exist, the need for a second grammar would increase if its considered that sufficient language change has taken place (other factors, such as the theoretical frame and the coverage of the former grammar, being equal).

## Usage Notes

Following CLDF standards, the GATA dataset^28^ is published as linked CSVW-files. It can easily be accessed either with CSV-reading applications or with designated tools developed from the CLDF-community. For example, designated programming packages for retrieving CLDF data have been developed for Python (https://github.com/cldf/pycldf) and R (https://github.com/SimonGreenhill/rcldf). The tabular CLDF format permits easy comparison with other CLDF-datasets. For example, it is possible to use the commandline interface of the pycldf-Python package to access the data or to create a SQlite database out of this dataset^29^. In general, the tabular-format makes it easy to reuse the data in various ways (associated with a large list of potential research questions), and with many different programs.

## Data Availability

The dataset is stored on Zenodo (10.5281/zenodo.8250217)^28^ and curated on Github (https://github.com/cldf-datasets/gata). The current release of the repository is Version 1.0.0 and was peer-reviewed in 2023. All data is available under a CC-BY 4.0 license. All scripts that have been used during the pre-processing of the data are made available within the repository. Specifically, Python-scripts were used after the manual annotation of the data for the standardization of all annotations, as well as for the aggregation of the individual spreadsheets. The script that was used for the conversion into CLDF is also part of this repository.
